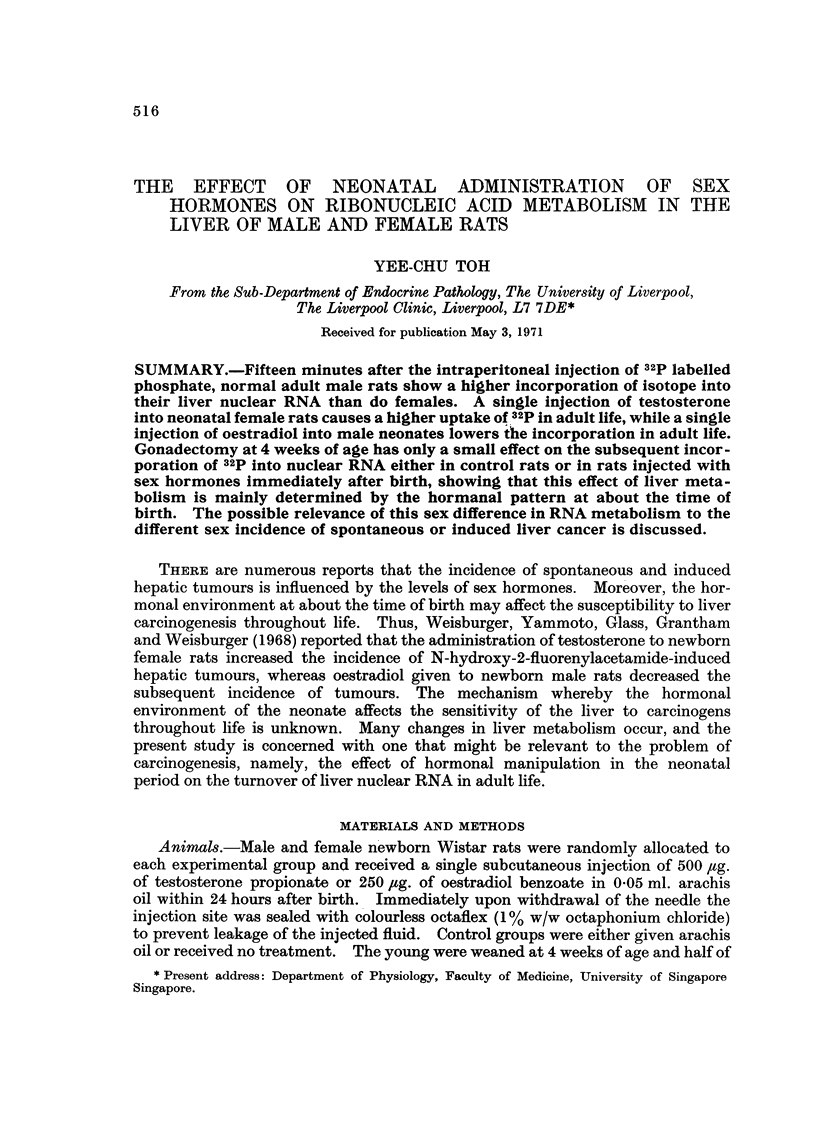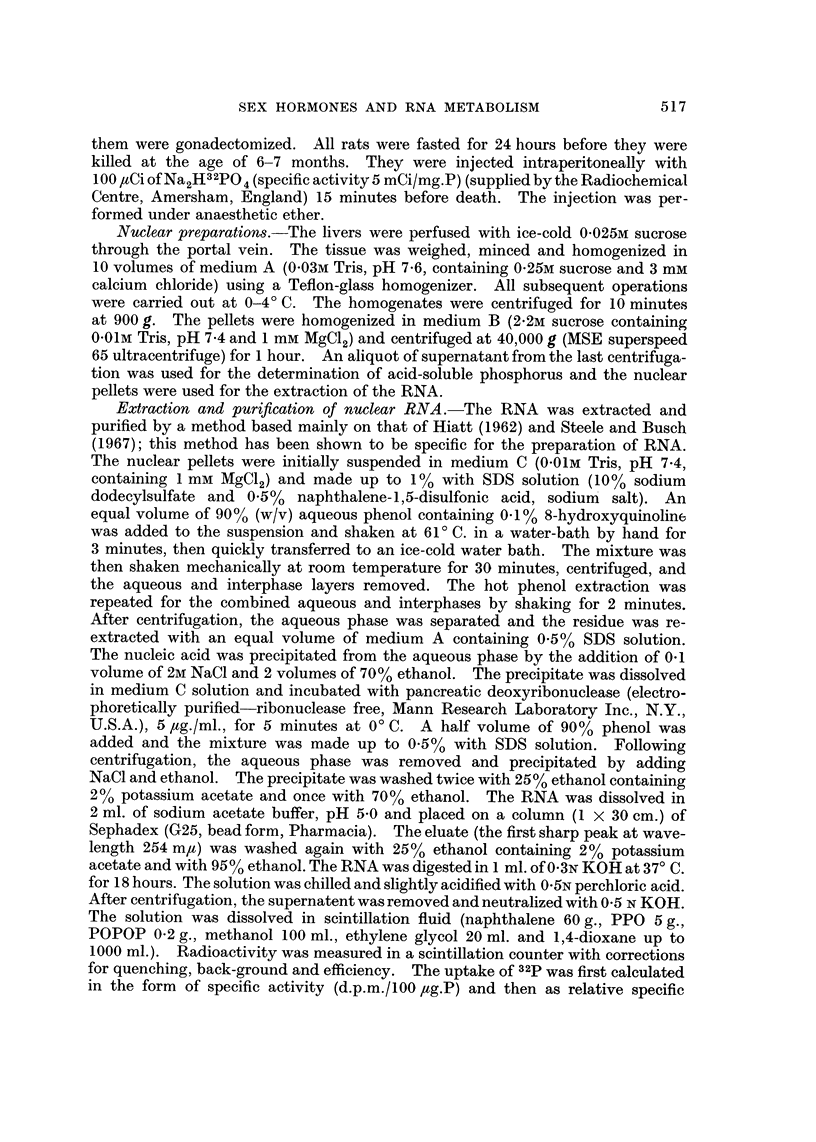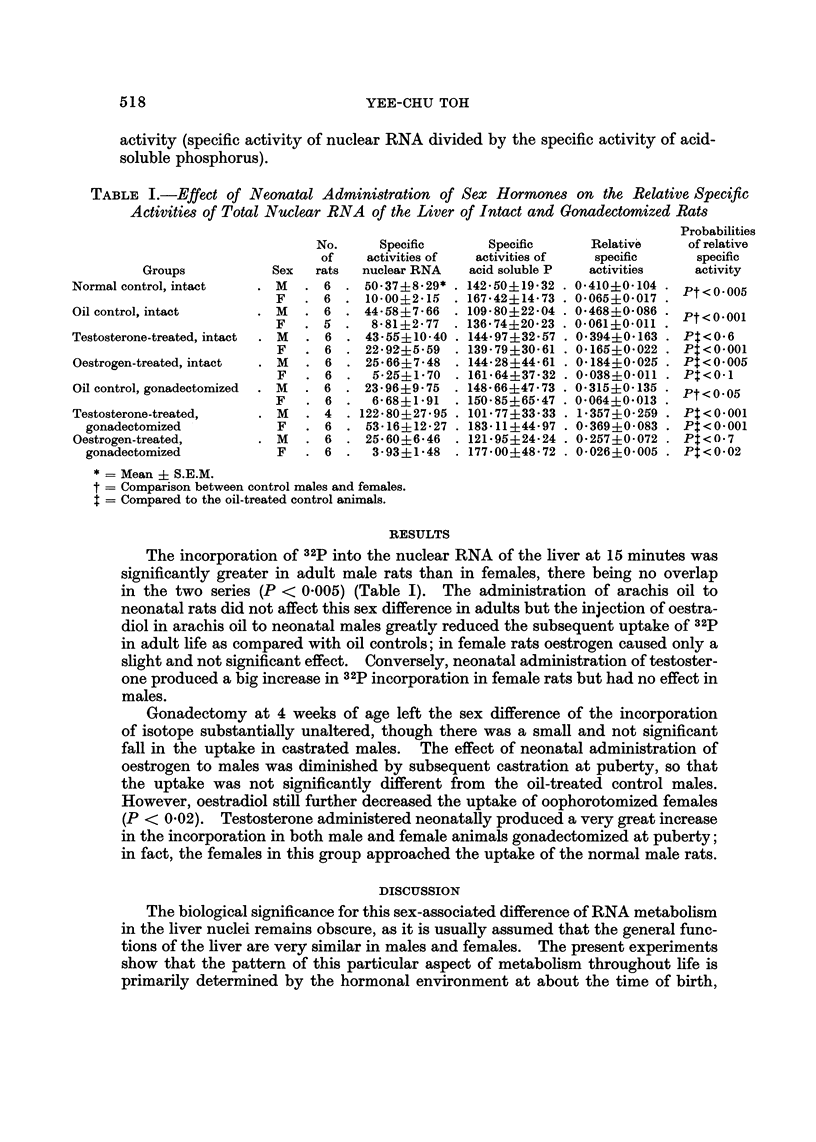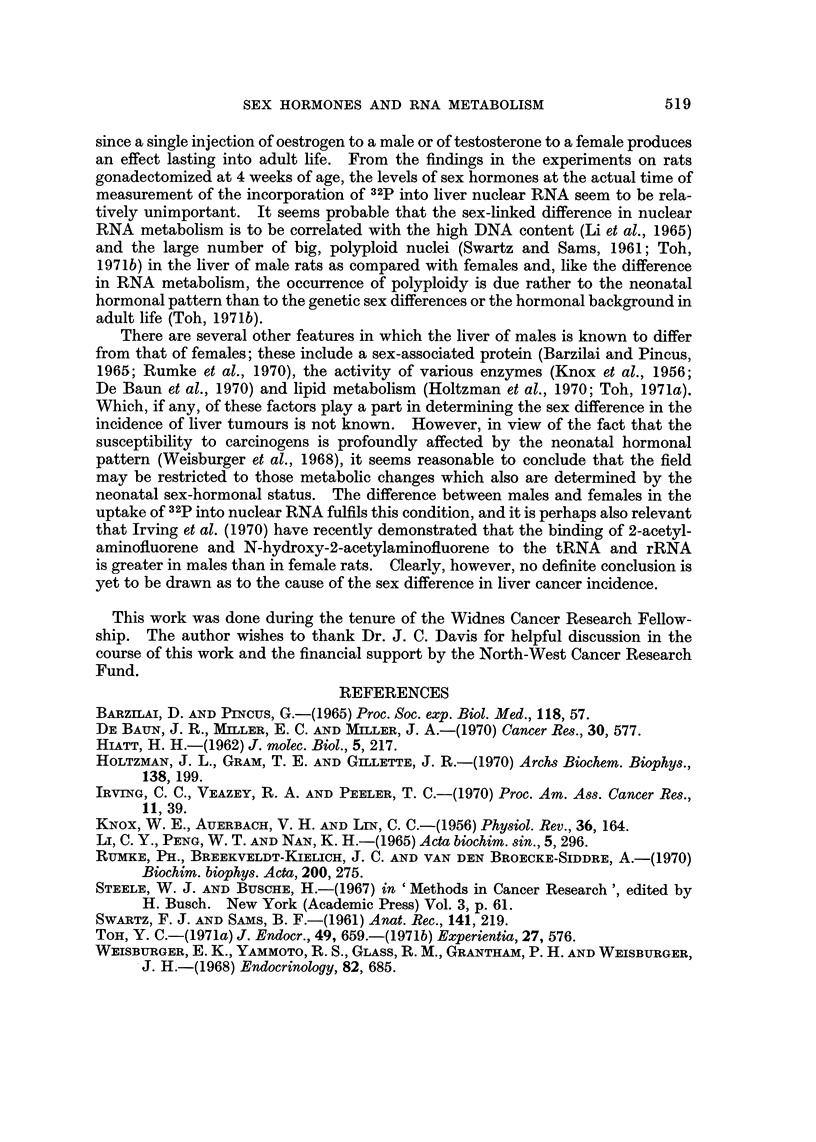# The Effect of Neonatal Administration of Sex Hormones on Ribonucleic Acid Metabolism in the Liver of Male and Female Rats

**DOI:** 10.1038/bjc.1971.65

**Published:** 1971-09

**Authors:** Yee-Chu Toh

## Abstract

Fifteen minutes after the intraperitoneal injection of ^32^P labelled phosphate, normal adult male rats show a higher incorporation of isotope into their liver nuclear RNA than do females. A single injection of testosterone into neonatal female rats causes a higher uptake of ^32^P in adult life, while a single injection of oestradiol into male neonates lowers the incorporation in adult life. Gonadectomy at 4 weeks of age has only a small effect on the subsequent incorporation of ^32^P into nuclear RNA either in control rats or in rats injected with sex hormones immediately after birth, showing that this effect of liver metabolism is mainly determined by the hormanal pattern at about the time of birth. The possible relevance of this sex difference in RNA metabolism to the different sex incidence of spontaneous or induced liver cancer is discussed.


### THE EFFECT OF NEONATAL ADMINISTRATION OF SEX HORMONES ON RIBONUCLEIC ACID METABOLISM IN THE LIVER OF MALE AND FEMALE RATS

YEE-CHU TOH

*From the Sub-Department of Endocrine Pathology, The University of Liverpool, The Liverpool Clinic, Liverpool, L7 7DE**

Received for publication May 3, 1971

**SUMMARY.—Fifteen minutes after the intraperitoneal injection of $^{32}P$ labelled phosphate, normal adult male rats show a higher incorporation of isotope into their liver nuclear RNA than do females. A single injection of testosterone into neonatal female rats causes a higher uptake of $^{32}P$ in adult life, while a single injection of oestradiol into male neonates lowers the incorporation in adult life. Gonadectomy at 4 weeks of age has only a small effect on the subsequent incorporation of $^{32}P$ into nuclear RNA either in control rats or in rats injected with sex hormones immediately after birth, showing that this effect of liver metabolism is mainly determined by the hormanal pattern at about the time of birth. The possible relevance of this sex difference in RNA metabolism to the different sex incidence of spontaneous or induced liver cancer is discussed.**

THERE are numerous reports that the incidence of spontaneous and induced hepatic tumours is influenced by the levels of sex hormones. Moreover, the hormonal environment at about the time of birth may affect the susceptibility to liver carcinogenesis throughout life. Thus, Weisburger, Yammoto, Glass, Grantham and Weisburger (1968) reported that the administration of testosterone to newborn female rats increased the incidence of N-hydroxy-2-fluorenylacetamide-induced hepatic tumours, whereas oestradiol given to newborn male rats decreased the subsequent incidence of tumours. The mechanism whereby the hormonal environment of the neonate affects the sensitivity of the liver to carcinogens throughout life is unknown. Many changes in liver metabolism occur, and the present study is concerned with one that might be relevant to the problem of carcinogenesis, namely, the effect of hormonal manipulation in the neonatal period on the turnover of liver nuclear RNA in adult life.

### MATERIALS AND METHODS

*Animals.*—Male and female newborn Wistar rats were randomly allocated to each experimental group and received a single subcutaneous injection of 500 $\mu$g. of testosterone propionate or 250 $\mu$g. of oestradiol benzoate in 0·05 ml. arachis oil within 24 hours after birth. Immediately upon withdrawal of the needle the injection site was sealed with colourless octaflex (1% w/w octaphonium chloride) to prevent leakage of the injected fluid. Control groups were either given arachis oil or received no treatment. The young were weaned at 4 weeks of age and half of

* Present address: Department of Physiology, Faculty of Medicine, University of Singapore Singapore.

them were gonadectomized. All rats were fasted for 24 hours before they were killed at the age of 6–7 months. They were injected intraperitoneally with 100 $\mu$Ci of $Na_2H^{32}PO_4$ (specific activity 5 mCi/mg.P) (supplied by the Radiochemical Centre, Amersham, England) 15 minutes before death. The injection was performed under anaesthetic ether.

*Nuclear preparations.*—The livers were perfused with ice-cold 0·025M sucrose through the portal vein. The tissue was weighed, minced and homogenized in 10 volumes of medium A (0·03M Tris, pH 7·6, containing 0·25M sucrose and 3 mM calcium chloride) using a Teflon-glass homogenizer. All subsequent operations were carried out at 0–4° C. The homogenates were centrifuged for 10 minutes at 900 *g*. The pellets were homogenized in medium B (2·2M sucrose containing 0·01M Tris, pH 7·4 and 1 mM $MgCl_2$) and centrifuged at 40,000 *g* (MSE superspeed 65 ultracentrifuge) for 1 hour. An aliquot of supernatant from the last centrifugation was used for the determination of acid-soluble phosphorus and the nuclear pellets were used for the extraction of the RNA.

*Extraction and purification of nuclear RNA.*—The RNA was extracted and purified by a method based mainly on that of Hiatt (1962) and Steele and Busch (1967); this method has been shown to be specific for the preparation of RNA. The nuclear pellets were initially suspended in medium C (0·01M Tris, pH 7·4, containing 1 mM $MgCl_2$) and made up to 1% with SDS solution (10% sodium dodecylsulfate and 0·5% naphthalene-1,5-disulfonic acid, sodium salt). An equal volume of 90% (w/v) aqueous phenol containing 0·1% 8-hydroxyquinoline was added to the suspension and shaken at 61° C. in a water-bath by hand for 3 minutes, then quickly transferred to an ice-cold water bath. The mixture was then shaken mechanically at room temperature for 30 minutes, centrifuged, and the aqueous and interphase layers removed. The hot phenol extraction was repeated for the combined aqueous and interphases by shaking for 2 minutes. After centrifugation, the aqueous phase was separated and the residue was re-extracted with an equal volume of medium A containing 0·5% SDS solution. The nucleic acid was precipitated from the aqueous phase by the addition of 0·1 volume of 2M NaCl and 2 volumes of 70% ethanol. The precipitate was dissolved in medium C solution and incubated with pancreatic deoxyribonuclease (electrophoretically purified—ribonuclease free, Mann Research Laboratory Inc., N.Y., U.S.A.), 5 $\mu$g./ml., for 5 minutes at 0° C. A half volume of 90% phenol was added and the mixture was made up to 0·5% with SDS solution. Following centrifugation, the aqueous phase was removed and precipitated by adding NaCl and ethanol. The precipitate was washed twice with 25% ethanol containing 2% potassium acetate and once with 70% ethanol. The RNA was dissolved in 2 ml. of sodium acetate buffer, pH 5·0 and placed on a column (1 × 30 cm.) of Sephadex (G25, bead form, Pharmacia). The eluate (the first sharp peak at wavelength 254 m$\mu$) was washed again with 25% ethanol containing 2% potassium acetate and with 95% ethanol. The RNA was digested in 1 ml. of 0·3N KOH at 37° C. for 18 hours. The solution was chilled and slightly acidified with 0·5N perchloric acid. After centrifugation, the supernatent was removed and neutralized with 0·5 N KOH. The solution was dissolved in scintillation fluid (naphthalene 60 g., PPO 5 g., POPOP 0·2 g., methanol 100 ml., ethylene glycol 20 ml. and 1,4-dioxane up to 1000 ml.). Radioactivity was measured in a scintillation counter with corrections for quenching, back-ground and efficiency. The uptake of $^{32}P$ was first calculated in the form of specific activity (d.p.m./100 $\mu$g.P) and then as relative specific activity (specific activity of nuclear RNA divided by the specific activity of acid-soluble phosphorus).

TABLE I.—*Effect of Neonatal Administration of Sex Hormones on the Relative Specific Activities of Total Nuclear RNA of the Liver of Intact and Gonadectomized Rats*

| Groups | Sex | No. of rats | Specific activities of nuclear RNA | Specific activities of acid soluble P | Relative specific activities | Probabilities of relative specific activity |
|---|---|---|---|---|---|---|
| Normal control, intact | M | 6 | 50·37±8·29* | 142·50±19·32 | 0·410±0·104 | $P$† < 0·005 |
| | F | 6 | 10·00±2·15 | 167·42±14·73 | 0·065±0·017 | |
| Oil control, intact | M | 6 | 44·58±7·66 | 109·80±22·04 | 0·468±0·086 | $P$† < 0·001 |
| | F | 5 | 8·81±2·77 | 136·74±20·23 | 0·061±0·011 | |
| Testosterone-treated, intact | M | 6 | 43·55±10·40 | 144·97±32·57 | 0·394±0·163 | $P$‡ < 0·6 |
| | F | 6 | 22·92±5·59 | 139·79±30·61 | 0·165±0·022 | $P$‡ < 0·001 |
| Oestrogen-treated, intact | M | 6 | 25·66±7·48 | 144·28±44·61 | 0·184±0·025 | $P$‡ < 0·005 |
| | F | 6 | 5·25±1·70 | 161·64±37·32 | 0·038±0·011 | $P$‡ < 0·1 |
| Oil control, gonadectomized | M | 6 | 23·96±9·75 | 148·66±47·73 | 0·315±0·135 | $P$† < 0·05 |
| | F | 6 | 6·68±1·91 | 150·85±65·47 | 0·064±0·013 | |
| Testosterone-treated, gonadectomized | M | 4 | 122·80±27·95 | 101·77±33·33 | 1·357±0·259 | $P$‡ < 0·001 |
| | F | 6 | 53·16±12·27 | 183·11±44·97 | 0·369±0·083 | $P$‡ < 0·001 |
| Oestrogen-treated, gonadectomized | M | 6 | 25·60±6·46 | 121·95±24·24 | 0·257±0·072 | $P$‡ < 0·7 |
| | F | 6 | 3·93±1·48 | 177·00±48·72 | 0·026±0·005 | $P$‡ < 0·02 |

* = Mean ± S.E.M.
† = Comparison between control males and females.
‡ = Compared to the oil-treated control animals.

### RESULTS

The incorporation of $^{32}P$ into the nuclear RNA of the liver at 15 minutes was significantly greater in adult male rats than in females, there being no overlap in the two series ($P < 0{\cdot}005$) (Table I). The administration of arachis oil to neonatal rats did not affect this sex difference in adults but the injection of oestradiol in arachis oil to neonatal males greatly reduced the subsequent uptake of $^{32}P$ in adult life as compared with oil controls; in female rats oestrogen caused only a slight and not significant effect. Conversely, neonatal administration of testosterone produced a big increase in $^{32}P$ incorporation in female rats but had no effect in males.

Gonadectomy at 4 weeks of age left the sex difference of the incorporation of isotope substantially unaltered, though there was a small and not significant fall in the uptake in castrated males. The effect of neonatal administration of oestrogen to males was diminished by subsequent castration at puberty, so that the uptake was not significantly different from the oil-treated control males. However, oestradiol still further decreased the uptake of oophorotomized females ($P < 0{\cdot}02$). Testosterone administered neonatally produced a very great increase in the incorporation in both male and female animals gonadectomized at puberty; in fact, the females in this group approached the uptake of the normal male rats.

### DISCUSSION

The biological significance for this sex-associated difference of RNA metabolism in the liver nuclei remains obscure, as it is usually assumed that the general functions of the liver are very similar in males and females. The present experiments show that the pattern of this particular aspect of metabolism throughout life is primarily determined by the hormonal environment at about the time of birth, since a single injection of oestrogen to a male or of testosterone to a female produces an effect lasting into adult life. From the findings in the experiments on rats gonadectomized at 4 weeks of age, the levels of sex hormones at the actual time of measurement of the incorporation of $^{32}P$ into liver nuclear RNA seem to be relatively unimportant. It seems probable that the sex-linked difference in nuclear RNA metabolism is to be correlated with the high DNA content (Li *et al.*, 1965) and the large number of big, polyploid nuclei (Swartz and Sams, 1961; Toh, 1971*b*) in the liver of male rats as compared with females and, like the difference in RNA metabolism, the occurrence of polyploidy is due rather to the neonatal hormonal pattern than to the genetic sex differences or the hormonal background in adult life (Toh, 1971*b*).

There are several other features in which the liver of males is known to differ from that of females; these include a sex-associated protein (Barzilai and Pincus, 1965; Rumke *et al.*, 1970), the activity of various enzymes (Knox *et al.*, 1956; De Baun *et al.*, 1970) and lipid metabolism (Holtzman *et al.*, 1970; Toh, 1971*a*). Which, if any, of these factors play a part in determining the sex difference in the incidence of liver tumours is not known. However, in view of the fact that the susceptibility to carcinogens is profoundly affected by the neonatal hormonal pattern (Weisburger *et al.*, 1968), it seems reasonable to conclude that the field may be restricted to those metabolic changes which also are determined by the neonatal sex-hormonal status. The difference between males and females in the uptake of $^{32}P$ into nuclear RNA fulfils this condition, and it is perhaps also relevant that Irving *et al.* (1970) have recently demonstrated that the binding of 2-acetylaminofluorene and N-hydroxy-2-acetylaminofluorene to the tRNA and rRNA is greater in males than in female rats. Clearly, however, no definite conclusion is yet to be drawn as to the cause of the sex difference in liver cancer incidence.

This work was done during the tenure of the Widnes Cancer Research Fellowship. The author wishes to thank Dr. J. C. Davis for helpful discussion in the course of this work and the financial support by the North-West Cancer Research Fund.

### REFERENCES

Barzilai, D. and Pincus, G.—(1965) *Proc. Soc. exp. Biol. Med.*, **118**, 57.
De Baun, J. R., Miller, E. C. and Miller, J. A.—(1970) *Cancer Res.*, **30**, 577.
Hiatt, H. H.—(1962) *J. molec. Biol.*, **5**, 217.
Holtzman, J. L., Gram, T. E. and Gillette, J. R.—(1970) *Archs Biochem. Biophys.*, **138**, 199.
Irving, C. C., Veazey, R. A. and Peeler, T. C.—(1970) *Proc. Am. Ass. Cancer Res.*, **11**, 39.
Knox, W. E., Auerbach, V. H. and Lin, C. C.—(1956) *Physiol. Rev.*, **36**, 164.
Li, C. Y., Peng, W. T. and Nan, K. H.—(1965) *Acta biochim. sin.*, **5**, 296.
Rumke, Ph., Breekveldt-Kielich, J. C. and van den Broecke-Siddre, A.—(1970) *Biochim. biophys. Acta*, **200**, 275.
Steele, W. J. and Busche, H.—(1967) *in* ' Methods in Cancer Research ', edited by H. Busch. New York (Academic Press) Vol. 3, p. 61.
Swartz, F. J. and Sams, B. F.—(1961) *Anat. Rec.*, **141**, 219.
Toh, Y. C.—(1971*a*) *J. Endocr.*, **49**, 659.—(1971*b*) *Experientia*, **27**, 576.
Weisburger, E. K., Yammoto, R. S., Glass, R. M., Grantham, P. H. and Weisburger, J. H.—(1968) *Endocrinology*, **82**, 685.